# Amelioration of Experimental Autoimmune Encephalomyelitis in C57BL/6 Mice by Photobiomodulation Induced by 670 nm Light

**DOI:** 10.1371/journal.pone.0030655

**Published:** 2012-01-24

**Authors:** Kamaldeen A. Muili, Sandeep Gopalakrishnan, Stacy L. Meyer, Janis T. Eells, Jeri-Anne Lyons

**Affiliations:** Department of Health Sciences, College of Health Sciences, University of Wisconsin–Milwaukee, Milwaukee, Wisconsin, United States of America; City of Hope National Medical Center and Beckman Research Institute, United States of America

## Abstract

**Background:**

The approved immunomodulatory agents for the treatment of multiple sclerosis (MS) are only partially effective. It is thought that the combination of immunomodulatory and neuroprotective strategies is necessary to prevent or reverse disease progression. Irradiation with far red/near infrared light, termed photobiomodulation, is a therapeutic approach for inflammatory and neurodegenerative diseases. Data suggests that near-infrared light functions through neuroprotective and anti-inflammatory mechanisms. We sought to investigate the clinical effect of photobiomodulation in the Experimental Autoimmune Encephalomyelitis (EAE) model of multiple sclerosis.

**Methodology/Principal Findings:**

The clinical effect of photobiomodulation induced by 670 nm light was investigated in the C57BL/6 mouse model of EAE. Disease was induced with myelin oligodendrocyte glycoprotein (MOG) according to standard laboratory protocol. Mice received 670 nm light or no light treatment (sham) administered as suppression and treatment protocols. 670 nm light reduced disease severity with both protocols compared to sham treated mice. Disease amelioration was associated with down-regulation of proinflammatory cytokines (interferon-γ, tumor necrosis factor-α) and up-regulation of anti-inflammatory cytokines (IL-4, IL-10) *in vitro* and *in vivo*.

**Conclusion/Significance:**

These studies document the therapeutic potential of photobiomodulation with 670 nm light in the EAE model, in part through modulation of the immune response.

## Introduction

Multiple sclerosis (MS) is a chronic neurodegenerative disease characterized by demyelination, axonal degeneration and subsequent loss of motor function. MS is considered autoimmune in nature, mediated by myelin-reactive CD4^+^ T cells. Recent studies suggest mitochondrial dysfunction and oxidative stress contribute to chronic MS [1]–[3]. Currently approved therapies, including the β-Interferons, Copaxone, and monoclonal antibody therapies, target the immune response and are only partially effective [4]–[7]. Until therapeutic strategies addressing the ongoing demyelination and axonal damage are developed, treatment of MS will remain incomplete.

Experimental Autoimmune Encephalomyelitis (EAE) is a well-studied, CD4^+^ T-cell-mediated inflammatory demyelinating disease of the central nervous system (CNS) and serves as the primary animal model for MS [8]. Much of what is understood of the pathogenesis of MS, as well as many therapeutic advances are derived from studies using the EAE model. It is generally accepted that autoreactive, myelin-specific T cells are responsible for disease initiation. However, as has been noted in MS, recent studies suggest that oxidative/nitrosative stress play a key role in the pathogenesis of EAE. Studies by Qi et al. demonstrated nitrosylation of CNS proteins very early in the disease process, prior to any evidence of immune infiltration, and prevention of nitrosoxidative stress ameliorated clinical EAE [9], [10]. Studies by Dutta et al support a central role for oxidative/nitrosative stress in the axonal loss leading to permanent disability [2], [3].

Photobiomodulation (PBM) employing light in the far-red (FR) to near-infrared (NIR) range (630–1000 nm) with low-energy lasers or light-emitting diode (LED) arrays has shown a therapeutic effect in various clinical conditions. Far-red/near-infrared light has been used clinically for 30 years for the treatment of soft tissue injuries and to promote wound healing [11]–[13]. More recent studies have demonstrated the therapeutic potential of photobiomodulation with FR/NIR light in the treatment of chemotherapy or radiation-induced mucositis in bone marrow transplant patients [14], ischemic injury in the heart [15]–[17], and neurodegenerative diseases [18]–[23]. The therapeutic mechanism of photobiomodulation is believed to function through activation of cellular photoacceptors and subsequent activation of transcription factors leading to improved energy metabolism and mitochondrial function [24]–[26]. As MS pathogenesis is due to an interaction of chronic inflammatory processes and mitochondrial dysfunction, we hypothesized that FR/NIR light therapy would ameliorate disease pathogenesis in MOG_35–55_ peptide-induced EAE in C57BL/6 (B6) mice.

Previous studies indicate that the photoacceptor through which FR/NIR light mediates the observed effects is cytochrome C oxidase, a key molecule in the electron transport chain leading to production of ATP [26]–[28]. The action spectrum of FR/NIR light overlays the absorption spectrum of cytochrome C oxidase: the biological activity of FR/NIR light is most pronounced around 670 nm and 830 nm with a nadir in both spectra around 728 nm, mirroring the absorption spectrum of cytochrome C oxidase [26]. Based on previous studies characterizing the action spectrum of FR/NIR light and the absorption spectrum of cytochrome c oxidase, we investigated therapeutic potential of photobiomodulation by 670 nm light for the treatment of MS using the EAE model. Treatment with 670 nm light prior to or with the onset of clinical signs resulted in amelioration of clinical EAE. Subsequent studies revealed decreased expression of proinflammatory cytokines and increased expression of anti-inflammatory cytokines by lymph node cells exposed to 670 nm light compared to sham treatment. Similar results were observed in spinal cords isolated from mice over the course of EAE. Hence, our findings support the hypothesis that photobiomodulation induced by 670 nm light ameliorates disease severity in EAE.

## Results

### Treatment with 670 nm Light Modulates Cytokine Production by Lymph Node Cells

**Figure 1:**
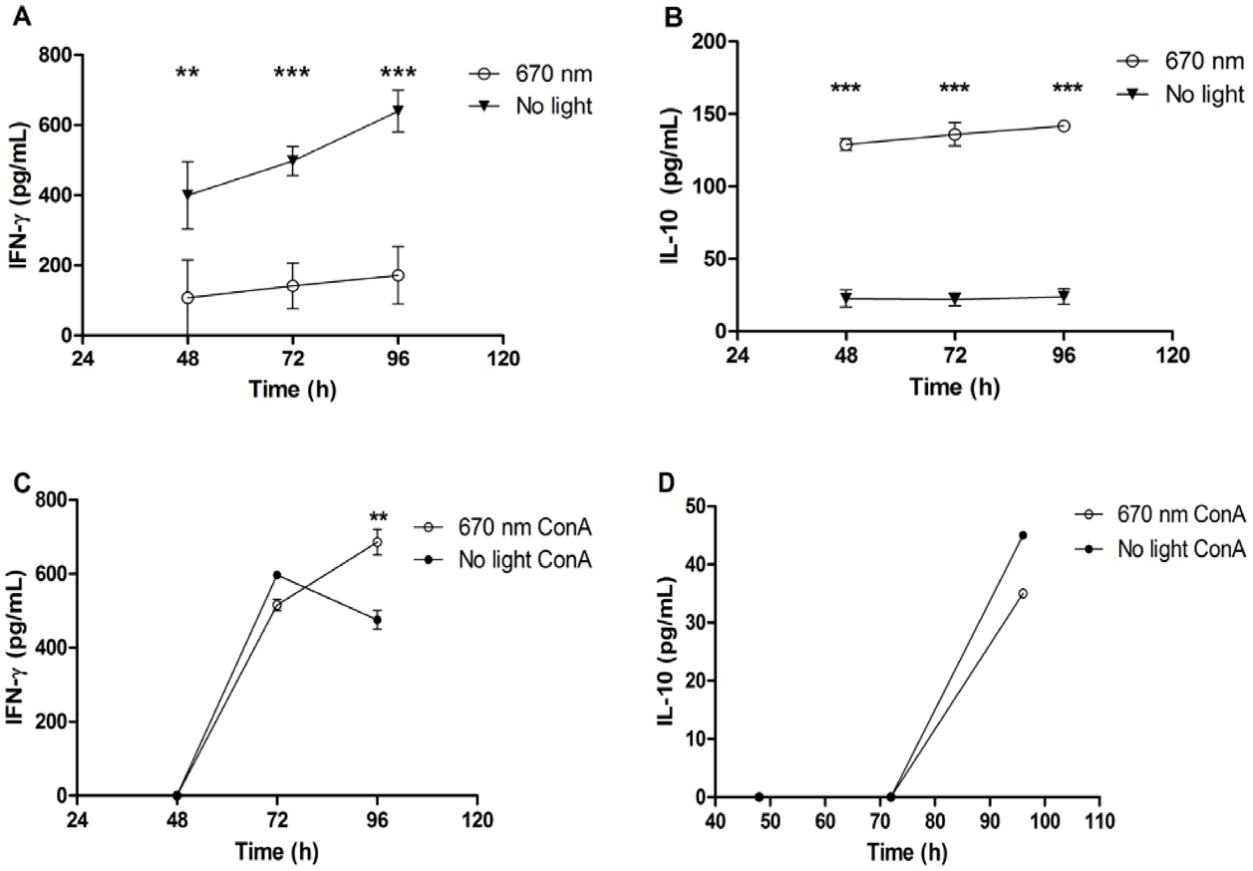
670 nm light modulates *in vitro* cytokine production by lymph node cells. Draining peripheral lymph nodes (PLN) were isolated at 10 days post immunization (dpi) from mice immunized with MOG_35–55_. Single cell suspensions were cultured (**A,B**) in the presence or absence of MOG_35–55_ or (**C,D**) with ConA (1 µg/mL) and were treated with 670 nm light (open symbols) or received no light treatment (closed symbols) once daily for 96 h. Cytokine ELISA was performed on supernatants for (**A,C**) IFN-γ and (**B,D**) IL-10. Data reported as background-subtracted (e.g., in the absence of MOG_35–55_ peptide) antigen-specific cytokine secretion. Data representative of 3 separate experiments. Error bars: SD. P<0.0001 by 2-way ANOVA. Multiple comparisons made via Bonferroni posttest; **P<0.01; ***P<0.001.

Previous studies demonstrated up-regulation of anti-inflammatory cytokines and down-regulation of inflammatory cytokines by FR/NIR light in animal models of acute and chronic inflammation [29], [30]. To determine if a similar effect is evident in antigen-specific immune responses, the direct effect of 670 nm light on cytokine production by antigen-primed immune cells was investigated. Peripheral lymph node cells isolated from MOG_35–55_-immunized mice were cultured with cognate peptide and received once daily treatment with 670 nm light or no light treatment for 96 h, and cytokine production was analyzed in supernatants. An antigen-specific effect of 670 nm light on cytokine production was noted, with down-regulation of IFN-γ, but up-regulation of IL-10 protein expression compared to cells not exposed to light treatment (Figure 1A, P<0.0001 and Figure 1B, P<0.0001). Stimulation of lymph node cells with the mitogenic lectin, Concanavalin A (ConA), resulted in cytokine expression similar to that observed by sham treated cells (Figure 1). A small but significant increase in IFN-γ was noted by ConA-stimulated lymph node cells treated with 670 nm light vs. sham treated cells at 96 h (Figure 1C). No significant difference in IL-10 expression by ConA stimulated cells was noted between treatment groups (Figure 1D).

### Amelioration of Clinical EAE by Treatment with 670 nm Light

**Figure 2:**
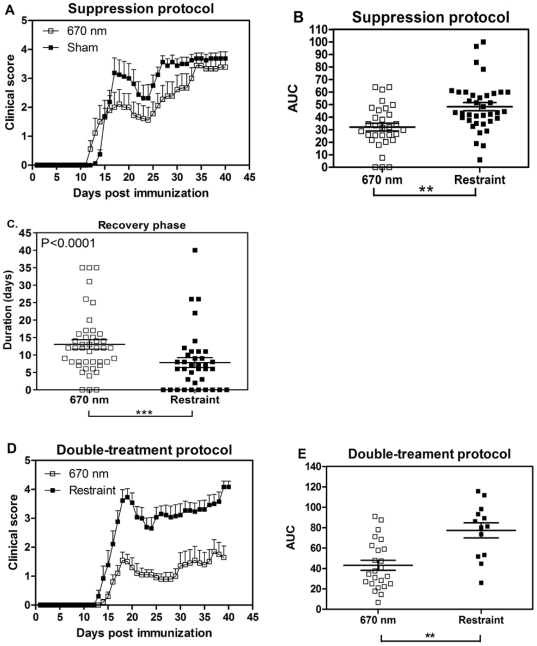
670 nm light treatment reduces mean clinical score and signs of MOG-induced EAE. EAE was induced with MOG_35–55_, and mice were treated with 670 nm light or no light (restraint) stress [sham]. (**A**) Mice treated with the suppression protocol. 670 nm light (-□-; n = 8) or sham (-▪-; n = 9). Representative 3 separate experiments with total n = 36 (670 nm light); n = 37 (sham). Error bars represent SEM. P<0.0001 by 2-way ANOVA. (**B**) Clinical course was plotted and the area under the curve (AUC) for all mice treated with the suppression protocol was determined and compared by 1-way ANOVA. Horizontal line: median value; Error bars: SEM. P<0.001. (**C**) duration of recovery for all mice treated with the suppression protocol was determined by counting the days of recovery from peak disease (decrease in 1 disease score) to the time of relapse (increase of 1 disease score); horizontal line: median value. (**D**) Mice were treated with 670 nm light (-□-; n = 10) or no light (restraint stress; -▪-; n = 7) using the double-treatment protocol. Representative of 3 separate experiments with a total n = 24 (670 nm) or 2 separate experiments n = 14 (restraint only-sham). Error bars represent SEM. P<0.0001 by 2-way ANOVA. (**E**) The AUC of clinical disease curves for all mice treated with the double treatment was determined; horizontal line: median value. Error bars: SEM; P = 0.0003 by 1-Way ANOVA. **P<0.01; ***P<0.001.

The immune modulation by 670 nm light observed *in vitro* would be expected to result in clinical improvement of EAE. The therapeutic potential of 670 nm light in the treatment of EAE/MS was initially investigated with a suppression protocol. Following immunization with MOG_35–55_, mice received 670 nm±30 nm light emitted by an LED array (75 cm^2^) at a fluence of 5 J/cm^2^ at the dorsal surface of the mouse beginning the day after immunization and continuing for 10 days. Disease was compared to sham treated animals subjected to restraint stress only. Animals receiving 670 nm light treatment presented with clinically less severe disease compared to animals exposed to restraint stress only (Figure 2A, B; P<0.0001 by 2-way ANOVA; Area Under the Curve (AUC) P<0.001 by 1-way ANOVA). The beneficial effect of 670 nm light was evident as a more sustained recovery in 670 nm treated animals compared to restraint stress-treated group (Figure 2C; P<0.01). This effect was temporary, as treated animals did eventually relapse to similar disease severity as sham treated animals (Figure 2A).

To address whether established disease could be ameliorated after the onset of clinical signs, 670 nm light therapy was initiated on the day of disease onset. Initial studies demonstrated a similar effect on disease course as was noted with the suppression protocol when animals received 670 nm light for 7 days beginning the day of disease onset (“Onset Protocol”), including eventual relapse to similar disease severity (not shown). In an attempt to improve the clinical efficacy of photobiomodulation by 670 nm light, the “Double-treatment Protocol” was developed. Treatment was initiated on the day of onset and continued for 7 days. This was followed by a 7 day “rest” period when animals received no light treatment and a subsequent 7 day treatment period. Comparison of disease severity in 670 nm light treated animals vs. those treated with restraint stress only revealed an improved clinical effect with this modified treatment protocol (Figure 2D; P<0.0001 by 2-way ANOVA; Figure 2E: AUC: P = 0.0003 by 1-way ANOVA). Furthermore, the observed treatment of 670 nm light was sustained throughout the duration of the experiment. Thus, photobiomodulation with 670 nm light showed a beneficial effect on the clinical course of actively induced EAE.

### Treatment with 670 nm Light Modulates Cytokine Production Over the Course of EAE

**Figure 3:**
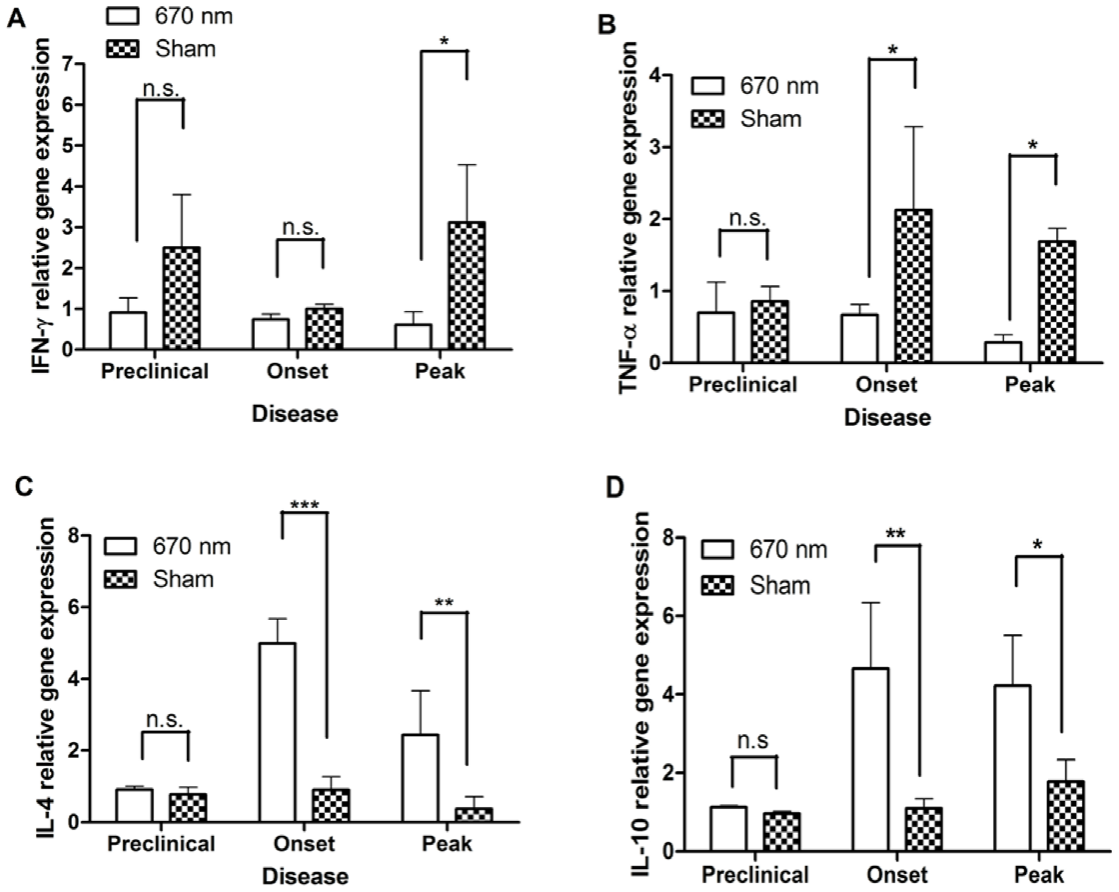
The suppression treatment protocol results in cytokine modulation within the CNS over the course of EAE. Spinal cords (SC) were isolated from MOG_35–55_-immunized mice treated with the suppression protocol and QPCR was performed to quantify cytokine gene expression. Relative gene expression was determined by the Paffl method (2000) Gene expression was normalized at each disease stage with sham treated mice. (**A**) INF-γ; (**B**) TNF-α; (**C**) IL-4; (**D**) IL-10. n = 4 mice per group. Error bars indicated SD between 2 independent experiments. Data analyzed 2-way ANOVA. Multiple comparisons by Bonferroni post-test. *P<0.05, **P<0.01, ***P<0.0001, n.s = not significant.

*In vitro* studies suggested that 670 nm light modulates antigen-specific cytokine production, shifting the balance of proinflammatory and anti-inflammatory cytokines in a manner that would be beneficial to the clinical course of EAE. To determine if a similar cytokine shift could be detected in treated mice over the course of EAE, gene expression was assayed by QPCR of cDNA from peripheral lymph nodes (PLN) and spinal cords (SC) isolated over the course of EAE from mice treated with 670 nm light or restraint stress by the suppression protocol. Similar to the in vitro observations, IFN-γ (Figure 3A) and TNF-α (Figure 3B) expression was significantly down-regulated in SC of EAE mice treated with 670 nm light compared with restraint-only treated animals. A concomitant up-regulation in IL-4 and IL-10 was noted in the spinal cord over the course of EAE (Figure 3C, D). Similar shifts in cytokine expression in the PLN at peak disease were also observed (data not shown).

**Figure 4:**
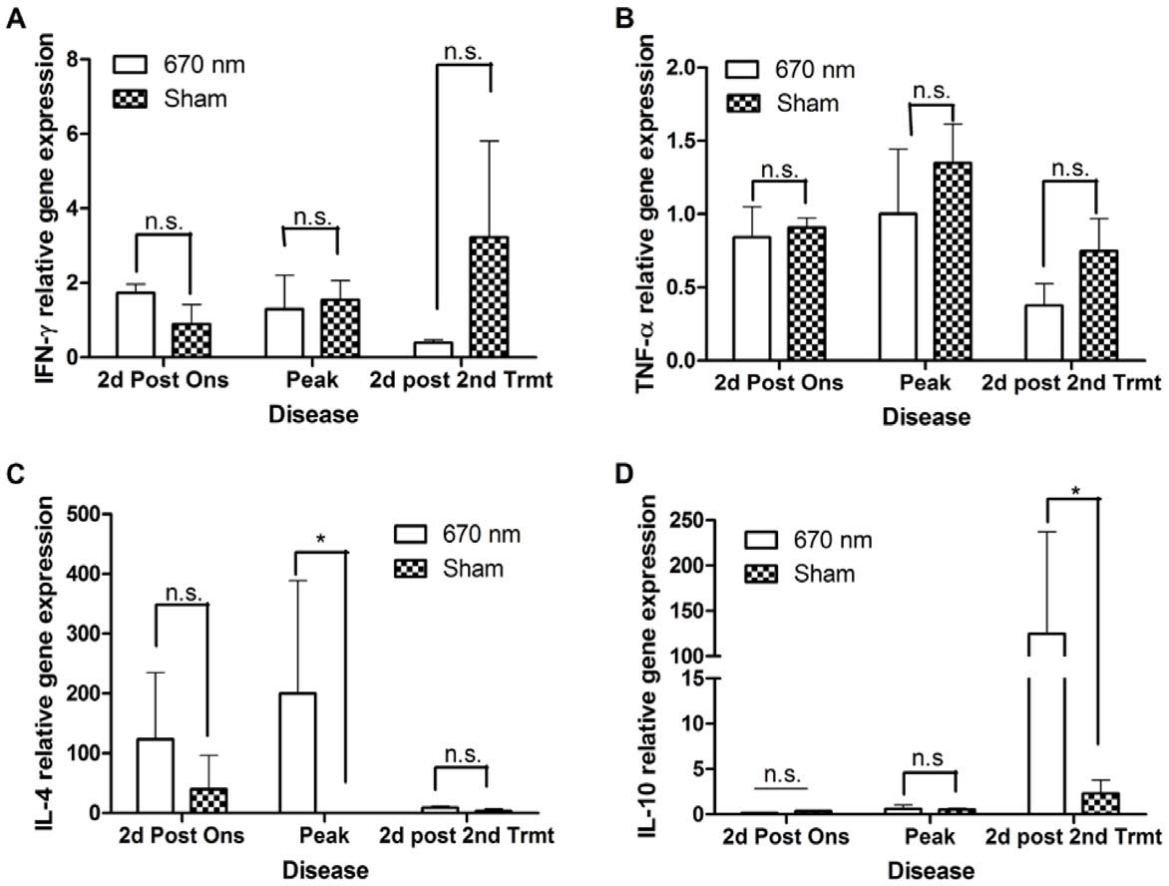
The Double-Treatment protocol results in up-regulation of anti-inflammatory cytokines within the CNS over the course of EAE. Spinal cords (SC) were isolated from MOG_35–55_-immunized mice treated with the double-treatment protocol and QPCR was performed to quantify cytokine gene expression. Gene expression was normalized at each disease stage with sham treated mice. (**A**) INF-γ; (**B**) TNF-α; (**C**) IL-4; (**D**) IL-10. n = 4 mice per group. Error bars indicate SD between 2 independent experiments. Data were analyzed by 2-way ANOVA. Multiple comparisons by Bonferroni post-test. *P<0.05, **P<0.01, ***P<0.0001, n.s. = not significant.

To determine whether similar cytokine modulation could be detected with the treatment of established disease, cytokine expression within the PLN and SC over the course of disease from animals treated with the double-treatment protocol was analyzed (Figure 4). A non-significant decrease in proinflammatory cytokine gene expression was noted in both PLN (data not shown) and SC (Figure 4A, B). However, increased anti-inflammatory cytokine expression was noted at both sites, with significant up-regulation of IL-4 at peak of acute disease (Figure 4C and data not shown) and significant up-regulation of IL-10 during chronic disease (Figure 4D and data not shown).

## Discussion

Multiple Sclerosis (MS) is a leading cause of neurologic disability in young adults. Disease pathogenesis is complex. Based on studies in the EAE model, disease is believed to be initiated by autoreactive CD4+ Th1 and/or Th17 lymphocytes specific for myelin proteins [31]. These cells are activated in the periphery by unknown mechanisms and infiltrate the CNS where they secrete proinflammatory cytokines (e.g., IFN-γ, TNF-α, IL-17) to initiate and propagate a proinflammatory response leading to demyelination of CNS axons by multiple mechanisms, including: cytokine-mediated demyelination by TNFα [32]; antibody-mediated mechanisms, ± complement [33]–[35]; macrophage/phagocytic mechanisms [36]–[38]; and oxidative stress [2], [3]. Ongoing insult leads to axonal loss and permanent disability [3], [39]. More recent studies indicate that disease progression is due to non-immune mechanism, suggesting a role for oxidative stress in the axonal loss occurring late in the disease process [2], [3].

The FDA-approved therapeutics for MS act through immunosuppressive or immunomodulatory mechanisms, targeting those processes important to the initiation of clinical activity associated with the relapsing/remitting phase of disease [4]–[6], [40]–[42]. These agents slow disease progression but do not prevent it, and are ineffective in secondary progressive or primary progressive disease, perhaps because they fail to provide a neuroprotective component. Thus, the development of new agents or adjunct therapies preventing demyelination or axonal loss, or promoting remyelination and oligodendrocyte function, is of great interest.

The therapeutic application of light to elicit a biological response has been studied using different modalities and different wavelengths. Various approaches, all involving introduction of an exogenous photosensitizer which is then activated to elicit a clinical effect, have been employed in a variety of systems, including the EAE model [43]–[47]. The biological response of ultraviolet light is well recognized [48]–[53], and has been studied in the EAE model [54]. Photobiomodulation, also referred to as Low Level Light Therapy (LLLT), as employed in the current studies, differs from these previous modalities in the use of FR/NIR light delivered in the absence of an exogenous photosensitive moiety (reviewed in [25]). Photobiomodulation has recently attracted increased attention. In particular, photobiomodulation performed with FR/NIR light has demonstrated efficacy in chronic wound healing in clinical and experimental systems [12], [13], [30] and for the treatment of neurodegenerative diseases [18]–[20], [22], including Parkinson's disease [22], [23], [55], retinitis pigmentosa [20], and stroke [56], [57]. As MS pathogenesis is proinflammatory and neurodegenerative, we investigated the therapeutic potential of FR/NIR light using the EAE model.

Data presented demonstrate that photobiomodulation by 670 nm light led to amelioration of the clinical severity of MOG_35–55_ induced EAE compared to sham-treated animals when treatment was initiated following immunization but prior to onset of clinical signs (e.g., suppression protocol) and by a single 7 day treatment protocol initiated on the day of disease onset (e.g., treatment protocol). Interestingly, the effect noted with these 2 protocols (e.g., decreased severity of the acute phase and increased duration of recovery) was similar. Furthermore, no effect on the onset of clinical signs was noted with the suppression protocol. These data suggest that there is no effect on the induction of the autoimmune response, but rather on the pathogenic mechanisms affecting the disease process.

The efficacy of the clinical effect of 670 nm light improved when a second treatment period was added (e.g., double-treatment protocol), leading to sustained clinical improvement in affected animals. The improved efficacy of the double-treatment protocol is explained by the current understanding of disease pathogenesis and the therapeutic mechanism of photobiomodulation. MS and EAE are, by nature, relapsing-remitting diseases, with initial attacks due to immunoregulatory mechanisms. While the B6 model of EAE employed here displays a more chronic phenotype, mice do often times recover partially from peak disease scores attained during the acute phase. Disease progression or sustained neurological deficit is thought to be due to oxidative damage to CNS axons. Photobiomodulation mediated by FR/NIR light is hypothesized to induce gene transcription, mediated by interaction of FR/NIR light with the cytochrome c oxidase photoacceptor [26]. Cytochrome c oxidase is an important participant in the electron transport chain, critical for the maintenance of mitochondrial function. The molecule binds, interchangeably, molecular oxygen and nitric oxide. Nitric oxide is known to mediate intracellular signal transduction cascades and communication between the mitochondrion and the nucleus [58]. Thus, interaction of FR/NIR light with cytochrome c oxidase is thought to lead to the release of nitric oxide, initiating signaling transduction cascades culminating in activation of transcription and translation of nuclear-encoded proteins, leading to the observed effects of photobiomodulation [17], [59]–[61]. A recent study by Chen et al suggests that the observed effects are at least in part mediated by the transcription factor, NFκB, and an increase in cellular ATP synthesis [62]. In the EAE model, these effects are apparent as down-regulation of pro-inflammatory mechanisms and up-regulation of anti-inflammatory mechanisms. Thus, gene induction by suppression and treatment protocols would be expected to be similar. However, with the cessation of the suppression protocol at or around the day of onset, gene transcription would eventually return to pre-treatment levels, thus abrogating the protective effect of FR/NIR light. On the other hand, the double-treatment protocol would be expected to sustain gene transcription and improve the clinical effect.

The structure of the double treatment protocol, with inclusion of a 7 day rest period, was developed with insight provided by other protocols not presented here. For example, early studies employed a protocol in which treatment was initiated the day after immunization and continued through the peak of disease. This protocol resulted in a worsening of clinical signs in 670 nm-treated animals compared to sham-treated mice. This could be due to the dual role of iNOS/nitric oxide in regulation of EAE and the immune response [63]–[68]. These studies indicated that nitric oxide is important in regulating T cell activation to neuroantigens during disease initiation [63] while playing a pathogenic role in ongoing disease [2], [64]. The 7 day rest period typically occurs during the remission/recovery phase of disease when the immune response responsible for relapse is being primed, and treatment is again initiated at or around the day of the first indications of relapse. Thus, this 7 day rest period may be providing an opportunity for important immunomodulatory functions of nitric oxide to occur, preventing excessive activation of autoreactive T cells, while subsequent treatment is down-regulating the pathogenic contribution of nitric oxide to disease progression. In support of this, more recent studies incorporating a third treatment period following a second 7 day rest demonstrated continued suppression of clinical signs in treated animals.

*In vitro* and *in vivo* experiments demonstrate modulation of pro-inflammatory vs. anti-inflammatory cytokines by 670 nm light in a manner consistent with the observed effects on disease course. It is interesting to note that immunomodulation appears to require signaling through antigen-specific mechanisms (i.e., the T cell receptor; TcR), as evidenced by a lack of cytokine modulation when cells are stimulated with ConA. ConA is a mitogenic lectin that activates T cells in a polyclonal manner via cross-linking of cell-surface β-glycans. It is generally used as a positive control for cell viability and the ability to respond to appropriate stimulatory signals. It does not interact with nor signal through the antigen-specific TcR and therefore activates different signal transduction cascades than would antigen-specific activation through the TcR and requisite co-stimulatory pathways (e.g., CD28). This observation could not only have relevance to the clinical application of 670 nm-mediated photobiomodulation but could also provide insight into the mechanisms behind the observed clinical effects.

The neuroprotection afforded by FR/NIR light noted in other systems is thought to be due to down-regulation of oxidative stress and improved cytoprotective mechanisms [25], [59], [69]. Indeed, ongoing studies suggest that similar mechanisms are involved in the observed effect of 670 nm light on the EAE disease course. However, given the autoimmune nature of MS and the described complications associated with monoclonal antibody therapies [70], as well as the known effects on chronic inflammation, it is important to understand the effect of FR/NIR light on the immune response. Understanding of the effect of FR/NIR light on immunity is limited [30], [71], [72]. Data from *in vitro* and *ex vivo* studies presented here demonstrate an immunomodulatory effect of 670 nm light on antigen-specific cytokine production, with down-regulation of proinflammatory cytokines, IFN-γ and TNF-α, and up-regulation of anti-inflammatory cytokines, IL-4 and IL-10. These cytokine changes would be expected to have a beneficial effect in the EAE model and on the clinical course of MS [4], [31], [73], [74].

Ongoing studies seek to develop photobiomodulation as an adjunct therapy for the treatment of MS, used in conjunction with the currently approved immunomodulatory agents. However, several questions need to be answered before translation into human system will be possible, including optimization of treatment wavelength, the nature of the treatment device, and the penetration of light to affected structures. A recent comprehensive review indicates photobiomodulation may achieve different effector functions, therapeutic or disease aggravation depending on choice of photobiomodulation parameters. These parameters include; 1) the optical properties of tissues, and 2) dositometry (i.e irradiance and dosing) [60]. A primary question that needs to be addressed before these questions can be answered is whether the observed effects are due to systemic effects or targeted (e.g., within the CNS) effects of light on the system. The total body irradiation employed here leaves this up to debate. The total body irradiation used here could result in systemic, transcutaneous effects as well as effects mediated by transmission of light through the optic nerve. On the one hand, the immunomodulation noted in the current studies may support a systemic effect, or at least an effect in the periphery as opposed to the CNS, as the disease-inducing T-cells must be activated in the periphery in order to traverse the blood brain barrier and cause disease [75], [76]. In addition, the immunomodulatory effects noted in the suppression protocol would have been primed in the lymph nodes. On the other hand, light would also be transmitted via the optic nerve or could directly penetrate the spinal cord. Ongoing studies demonstrate decreased oxidative stress and apoptosis within the spinal cord of treated animals. Furthermore, recent studies utilizing a similar LED device demonstrated that transmission of light via the optic nerve preserved CNS function following transection of the optic nerve [77]. These will be challenging questions to answer, and it is possible that both systemic and targeted mechanisms contribute to the observed clinical effects. Further studies and procurement of additional instrumentation will be necessary to address these questions, and the answers to these questions will be important to the translation of these studies to the human population.

Several studies have established the role of immune system in EAE and MS [31]. Recent studies have implicated other pathogenic mechanisms, including mitochondrial dysfunction and oxidative/nitrosative stress in EAE and MS [2], [3], [9], [10], [62]. Data presented here show that treatment with 670 nm light ameliorates disease severity in EAE, in part through immunomodulatory mechanisms. Ongoing studies further investigate the observed immune modulation and address the role of FR/NIR light in preservation of mitochondrial function and remediation of oxidative stress in the EAE model. These experiments suggest a combination of both mechanisms in the amelioration of EAE, indicating that photobiomodulation may be valuable as an adjunct therapy for the treatment of MS.

## Materials and Methods

### Animals

Specific pathogen-free female C57BL/6 (B6) WT mice were bred in-house from breeding pairs purchased from Jackson Laboratories (Bar Harbor, ME). Mice were maintained in micro-isolator cages in an AAALAC-accredited facility in accordance with University and NIH guidelines. All animals were supplied with food and water ad libitum and maintained on a 12 h light/dark schedule in a temperature and humidity-controlled environment.

### Antigens and EAE induction

The mouse MOG_35–55_ peptide (MEVGWYRSPFSRVVHLYRNGK) was synthesized and purity confirmed by HPLC (GenScript, Piscataway, NJ). EAE was induced in mature mice (6–8 weeks old) by immunization with 100 µg MOG_35–55_ peptide emulsified (1:1) in IFA with 300 µg *Mycobacterium tuberculosis*, strain H37RA (Bectin Dickinson, FranklinLakes, NJ). Each mouse received 0.05 mL emulsion (s.c.) at four sites. In addition, animals received pertussis toxin (300 ng i.p.; List Laboratories, Campbell, CA) at 0 h and 72 h post immunization. Animals were followed for the development of EAE and graded clinically on a scale of 0–5 by a blinded observer [0: healthy, no signs of EAE; 1: loss of tail tone; 2: hind limb weakness; 3: paresis or paralysis of one hind limb; 4: paralysis of both hind limbs; 5: dead or moribund].

### LED Treatment

Gallium/Aluminum/Arsenide (GaAlA) LED arrays (75 cm^2^) of 670 nm (LED bandwidth 25–30 nm at Full Width Max Power [FWHM]) were obtained from Quantum Devices (Barneveld, WI). Unanesthetized Mice were placed individually into a polypropylene restraint device (12.7×9×7.6 cm), and the LED array was positioned directly over the animal at a distance of 2 cm, covering the entire chamber and exposing the entire dorsal surface. Treatment consisted of once daily irradiation at 670 nm for 3 min, at a power intensity of 28 mW/cm^2^ (total power output: 2100 mW) and an energy density of 5 J/cm^2^ (375 J daily total). To determine the energy density at the level of the spinal cord, a small incision was made at the base of the tail, and a probe was inserted underneath the skin at the base of the brain, demonstrating an energy density of 12 mJ/cm^2^ (432 mJ total energy administered daily) at the level of the spinal cord. As indicated, “restraint only” stress was employed, in which mice were placed in the restraint device for 3 min but not exposed to light. The Suppression Protocol consisted of once daily treatment for 10 consecutive days starting 24 h post immunization, resulting in a total of 4320 J at the level of the spinal cord administered to the animal over the course of treatment. Treatment protocols were performed as the “Onset Protocol”, consisting of once daily treatment for 7 consecutive days beginning the day of onset of clinical signs (clinical score = 1.0; 3024 J total energy administered), and the “Double Treatment Protocol”, consisting of once daily treatments initiated on the day of onset of clinical signs for 7 days, followed by 7 days rest, and a subsequent 7 day treatment period (6048 J total energy administered). Clinical disease was followed for an additional 7 days following cessation of the second treatment period.

### Real-time PCR

Mice were anesthetized with ketamine cocktail and perfused with 60 mL cold PBS via cardiac puncture. Total RNA was isolated from the spinal cord (SC) and the draining peripheral lymph nodes (PLN; e.g., popliteal, brachial and axillary lymph nodes.) using the Trizol method according to manufacturer's instructions (Invitrogen, Carlsbad, CA). RNA was further purified utilizing the RNEasy kit, with genomic DNA elimination columns and subsequent on-column DNase treatment to eliminate genomic DNA contamination (Qiagen, Valencia, CA). RNA (2 µg) was reverse transcribed using oligo dT primer (Operon, Huntsville, AL) and MMLV reverse transcriptase (Promega, Madison, WI) according to the manufacturer's instructions. Probe-based quantitative real-time PCR (QPCR) was performed for IFN-γ, TNF-α, IL-10, IL-4. β-actin was included as the housekeeping gene, and water blanks were included in all experiments. Primer/Probe sets were designed using the Universal Probe Design software (Roche Applied Science, Indianapolis, IN). Primers were designed to span introns, when possible. Primers were purchased from Sigma (St. Louis, MO), and probes were purchased from Roche Applied Science. Amplifications were performed using TaqMan®Universal PCR Master Mix (Roche Applied Science) on a SmartCycler (Cepheid, Sunnyvale, California), programmed for 95°C for 10 min, followed by 40 cycles of 95°C for 15 s and 60°C for 1 min, with detection of signal during the annealing/amplification segment. Results were calculated via the Pfaffl method (Pfaffl 2001) and are expressed as fold change in 670 nm treated mice relative to gene expression in samples isolated from sham treated animals at the same disease stage.

### Cytokine ELISA

Draining PLN were pooled from 6 mice 10 days post immunization (dpi) with 100 µg MOG_35–55_. Single cell suspensions were prepared, and cells (2.5×10^6^/mL) were cultured in supplemented RPMI 1640 [10% FCS, penicillin (100 U/mL)/streptomycin (100 µg/mL), L-glutamate (2 mM), Sodium pyruvate (0.1 mM), 2-mecarptoethanol (50 mM)] in 96-well flat-bottom plates (BD Biosciences, San Jose, California, USA) in the presence or absence of 10 µg/mL MOG_35–55_ peptide. Stimulation of cells with concanavalin A (ConA; 1 µg/mL) served as a positive control for cell viability. Cell cultures were treated once daily with 670 nm light or no light treatment beginning 2 h after plating and continuing at 24 h intervals for 96 h. Cell culture supernatants were isolated at 48 h, 72 h, 96 h, and 120 h and analyzed for IFNγ (<2.0 pg/mL detection limit) and IL-10 (<4.0 pg/mL detection limit) utilizing Quantikine kits according to manufacturer's instructions (R&D Systems, Minneapolis, MN). Data is reported as background-corrected (e.g., in the absence of peptide), antigen-specific cytokine secretion.

### Data Analysis

Data were analyzed and statistical analyses were performed using GraphPad Prism 4.0 (GraphPad, La Jolla, CA USA). Clinical course was compared by 2-way ANOVA. AUC analysis was compared by 1-way ANOVA (Kruskal-Wallis test); multiple comparisons performed by Dunn's Multiple Comparisons test or Bonferroni correction, as indicated. Mann-Whitney U-test and Student's t-test performed, as appropriate. P<0.05 was considered significant.

## References

[1] Bjartmar C, Trapp BD, Cook SD (2001). Axonal Injury in Multiple Sclerosis.. Handbook of Multiple Sclerosis.

[2] Dutta R, McDonough J, Yin X, Peterson J, Chang A (2006). Mitochondrial dysfunction as a cause of axonal degeneration in multiple sclersosis patieints.. Annals of Neurology.

[3] Dutta R, Trapp BD (2007). Pathogenesis of axonal and neuronal damage in multiple sclerosis.. Neurology.

[4] Duda PW, Schmied MC, Cook SL, Krieger JI, Hafler DA (2000). Glatiramer acetate (Copaxone) induces degenerate Th2-polarized immune responses in patients with multiple sclerosis.. Journal of Clinical Investigation.

[5] Johnson KP (2007). Natalizumab (Tysabri) treatment for relapsing multiple sclerosis.. Neurologist.

[6] Lou J, Gasche Y, Zheng L, Giroud C, Morel P (1999). Interferon-beta inhibits activated leukocyte migration through human brain microvascular endothelial cell monolayer.. Laboratory Investigation.

[7] Prinz M, Schmidt H, Mildner A, Knobeloch KP, Hanisch UK (2008). Distinct and nonredundant in vivo functions of IFNAR on myeloid cells limit autoimmunity in the central nervous system.[see comment].. Immunity.

[8] Croxford AL, Kurschus FC, Waisman A (2011). Mouse models for multiple sclerosis: historical facts and future implications.. Biochimica et Biophysica ACTA.

[9] Qi X, Lewin AS, Sun L, Hauswirth WW, Guy J (2006). Mitochondrial protein nitration primes neurodegeneration in experimental autoimmune encephalomyelitis.. Journal of Biological Chemistry.

[10] Qi X, Lewin AS, Sun L, Hauswirth WW, Guy J (2007). Suppression of mitochondrial oxidative stress provides long-term neuroprotection in Experimental Autoimmune Encephalomyelitis.. Investigative Ophthalmology & Vision Science.

[11] Lyons RF, Abergel RP, White RA, Dwyer RM, Castel JC (1987). Biostimulation of wound healing in vivo by a helium-neon laser.. Annals of Plastic Surgery.

[12] Whelan HT, Buchmann EV, Dhokalia A, Kane MP, Whelan NT (2003). Effect of NASA light-emitting diode irradiation on molecular changes for wound healing in diabetic mice.. Journal of Clinical Laser Medicine & Surgery.

[13] Whelan HT, Smits RL, Buchmann EV, Whelan NT, Turner SG (2001). Effect of NASA light-emitting diode (LED) irradiation on wound healing.. Journal of Clinical Laser Medicine & Surgery.

[14] Whelan HT, Connelly JF, Hodgson BD, Barbeau L, Post AC (2002). NASA light-emitting diodes for the prevention of oral mucositis in pediatric bone marrow transplant patients.. Journal of Clinical Laser Medicine & Surgery.

[15] Oron U, Yaakobi T, Oron A, Hayam G, Gepstein L (2001). Attenuation of infarct size in rats and dogs after myocardial infarction by low-energy laser irradiation.. Lasers Surg Med.

[16] Oron U, Yaakobi T, Oron A, Mordechovitz D, Shofti R (2001). Low-energy laser irradiation reduces formation of scar tissue after myocardial infarction in rats and dogs.. Circulation.

[17] Zhang R, Mio Y, Pratt PF, Lohr N, Warltier DC (2009). Near infrared light protects cardiomyocytes from hypoxia and reoxygenation injury by a nitric oxide dependent mechanism.. Journal of Molecular Cellular Cardiology.

[18] DeSmet KD, Paz DA, Corry JJ, Eells JT, Wong-Riley MTT (2006). Clinical and experimental applications of NIR-LED photobiomodulation.. Photomedicine and Laser Surgery.

[19] Eells JT, DeSmet KD, Kirk DK, Wong-Riley MT, Whelan HT, Tata D, Waynant RW (2008). Photobiomodulation in the treateatment of retinal injury and retinal degenerative diseases.. Light Activated Tissue Regeneration and Therapy.

[20] Eells JT, Kirk DK, Cribb J, Valter K, DeSmet KD (2006). Near–Infrared Light Therapy for Retinitis Pigmentosa.. Investigative Ophthalmology & Vision Science.

[21] Eells JT, Whelan HT, Salomao S, Berezovsky A, Paula H (2004). 670 nm LED Treatment of Affected Carriers of the 11778 Leber's Hereditary Optic Neuropathy (LHON) Mutation in Brazil.. Invest Ophthalmol Vis Sci.

[22] Ying R, Liang HL, Whelan HT, Eells JT, Wong-Riley MTT (2008). Pretreatment with near-infrared light via light-emitting diode provides added benefit against rotenone and MPP+ induced neurotoxicity.. Brain Research.

[23] Liang HL, Whelan HT, Eells JT, Wong-Riley MT (2008). Near-infrared light via light-emitting diode treatment is therapeutic against rotenone- and 1-methyl-4-phenylpyridinium ion-induced neurotoxicity.. Neuroscience.

[24] Eells JT, Wong-Riley MTT, VerHoeve J, Henry MM, Buchman EV (2004). Mitochondrial Signal Transduction in Accelerated Wound and Retinal Healing by Near-Infrared Light Therapy.. Mitochondrion.

[25] Hamblin MR, Demidova TN (2006). Mechanisms of Low Level Light Therapy.. Procedings of SPIE.

[26] Wong-Riley MTT, Liang HL, Eells JT, Chance B, Henry MM (2005). Photobiomodulation directly benefits primary neurons functionally inactivated by toxins: Role of cytochrome c oxidase.. Journal of Biological Chemistry.

[27] Wong-Riley MTT, Bai X, Buchman E, Whelan HT (2001). Ligh-emitting diode treatment reverses the effect of TTX on cytochrome oxidase in neurons.. NeuroReport.

[28] Karu TI, Pyatibrat LV, Kalendo GS (2004). Photobiological modulation of cell attachment via cytochrome c oxidase.. Photochem Photobiol Sci.

[29] Albertini R, Villaverde AB, Aimbire F, Bjordal JM, Brugnera A (2008). Cytokine mRNA expression is decreased in the subplantar muscle of rat paw subjected to carrageenan-induced inflammation after low-level laser therapy.. Photomedicine and Laser Surgery.

[30] Arany PR, Nayak RS, Halikerimath S, Limaye AM, Kale AD (2007). Activation of latent TGF-beta1 levels in laser-enhanced oral wound healing.. Wound Repair & Regeneration.

[31] Sospedra M, Martin R (2005). Immunology of Multiple Sclerosis.. Annual Review of Immunology.

[32] Caminero A, Comabella M, Montalban X (2011). Tumor necrosis factor alpha (TNF-α), anti-TNF-α and demyelination revisited: An ongoing story.. Journal of Neuroimmunology.

[33] Schluesener HJ, Sobel RA, Linington C, Weiner HL (1987). A monoclonal antibody against a myelin oligodendrocyte glycoprotein induces relapses and demyelination in central nervous system autoimmune disease.. Journal of Immunology.

[34] Genain CP, Nguyen M-H, Letvin NL, Pearl R, Davis RL (1995). Antibody facilitation of multiple sclerosis-like lesions in a nonhuman primate.. Journal of Clinical Investigation.

[35] Weber MS, Hemmer B, Cepok S (2011). The role of antibodies in multiple sclerosis.. Biochimica et Biophysica ACTA.

[36] Craner MJ, Damarjian TG, Liu S, Hains BC, Lo AC (2005). Sodium channels contribute to microglia/macrophage activation and function in EAE and MS.. Glia.

[37] Bauer J, Sminia T, Wouterlood FG, Dijkstra CD (1994). Phagocytic activity of macrophages and microglial cells during the course of acute and chronic relapsing experimental autoimmune encephalomyelitis.. Journal of Neuroscience Research.

[38] Hendriks JJA, de Vries HE, van der Pol SMA, van den Berg TK, van Tol EAF (2003). Flavonoids inhibit myelin phagocytosis by macrophages; a structure-activity relationship study.. Biochemical Pharmacology.

[39] Andrews HE, Nichols PP, Bates D, Turnbull DM (2005). Mitochondrial dysfunction plays a key role in progressive axonal loss in multiple sclerosis.. Medical Hypotheses.

[40] Aharoni R, Eilam R, Domev H, Labunskay G, Sela M (2005). The immunomodulator glatiramer acetate augments the expression of neurotrophic factors in brains of experimental autoimmune encephalomyelitis mice.. Procedings of the National Academy of Sciences.

[41] Hussein Y, Sanna A, Soderstrom M, Link H, Huang Y-M (2001). Glatiramer acetate and IFN-beta act on dendritic cells in multiple sclerosis.. Journal of Neuroimmunology.

[42] Stuve O, Dooley NP, Uhm JH, Antel JP, Williams G (1996). Interferon beta-1b decreases the migration of T lymphocytes in vitro: Effects on matrix metalloproteinase-9.. Annals of Neurology.

[43] Mitton D, Ackroyd R (2008). A brief overview of photodynamic therapy in Europe.. Photodiagnosis & Photodynamic Therapy.

[44] Ackroyd R, Kelty C, Brown N, Reed M (2001). The history of photodetection and photodynamic therapy.. Photochemistry & Photobiology.

[45] Cavaletti G, Perseghin P, Buscemi F, Dassi M, Oggioni N (2001). Immunomodulating effects of extracorporeal photochemotherapy in rat experimental allergic encephalomyelitis.. International Journal of Tissue Reactions.

[46] Chabannes D, Besnier DP, Esnault VLM (2002). Photopheresis affects the course of experimental allergic encephalomyelitis in Lewis rat.. Photodermatology, Photoimmunology & Photomedicine.

[47] Leong S, Chan AH, Levy JG, Hunt DW (1996). Transcutaneous photodynamic therapy alters the development of an adoptively transferred form of murine experimental autoimmune encephalomyelitis.. Photochemistry & Photobiology.

[48] el-Ghorr AA, Norval M (1997). The role of interleukin-4 in ultraviolet B light-induced immunosuppression.. Immunology.

[49] Vermeer BJ, Hurks M (1994). The clinical relevance of immunosuppression by UV irradiation.. Journal of Photochemistry & Photobiology B - Biology.

[50] Walterscheid JP, Nghiem DX, Ullrich SE (2002). Determining the role of cytokines in UV-induced immunomodulation.. Methods (Duluth).

[51] Ullrich SE, Schmitt DA (2000). The role of cytokines in UV-induced systemic immune suppression.. Journal of Dermatological Science.

[52] Ullrich SE, Nghiem DX, Khaskina P (2007). Suppression of an established immune response by UVA– critical role for mast cells.. Photochemistry & Photobiology.

[53] Ullrich SE, McIntyre BW, Rivas JM (1990). Suppression of the immune response to alloantigen by factors released from ultraviolet-irradiated keratinocytes.. Journal of Immunology.

[54] Hauser SL, Weiner HL, Che M, Shapiro ME, Gilles F (1984). Prevention of experimental allergic encephalomyelitis (EAE) in the SJL/J mouse by whole body ultraviolet irradiation.. Journal of Immunology.

[55] Shaw VE, Spana S, Ashkan K, Benabid A-L, Stone J (2010). Neuroprotection of Midbrain Dopaminergic Cells in MPTP-Treated Mice after Near-infrared Light Treatment.. Journal of Comparative Neurology.

[56] Lampl Y, Zivin JA, Fisher M, Lew R, Welin L (2007). Infrared Laser Therapy for Ischemic Stroke: A New Treatment Strategy: Results of the NeuroThera Effectiveness and Safety Trial-1 (NEST-1).. Stroke.

[57] Oron A, Oron U, Chen J, Eilam A, Zhang C (2006). Low-Level Laser Therapy Applied Transcranially to Rats After Induction of Stroke Significantly Reduces Long-Term Neurological Deficits.. Stroke.

[58] Nathan C (2003). Specificity of a third kind: reactive oxygen and nitrogen intermediates in cell signaling.. Journal of Clinical Investigation.

[59] Huang Y-Y, Chen AC-H, Carroll JD, Hamblin MR (2009). Biphasic Dose Response in Low Level Light Therapy.. Dose-Respone.

[60] Chung H, Dai T, Sharma SK, Huang YY, Caroll JD (2011). The Nuts and Bolts of Low-level Laser (Light) Therapy.. Annals of Biomedical Engineering.

[61] Poyton RO, Ball KA (2011). Therapeutic photobiomodulation: nitric oxide and a novel function of mitochondrial cytochrome c oxidase.. Discovery Medicine.

[62] Chen AC, Arany PR, Huang YY, Tomkinson EM, Sharma SK (2011). Low-level laser therapy activates NF-kB via generation of reactive oxygen species in mouse embyronic fibroblasts.. PLoS ONE [Electronic Resource].

[63] Cross AH, Keeling RM, Goorha S, San M, Rodi C (1996). Inducible nitric oxide synthase gene expression and enzyme activity correlate with disease activity in adoptively-transferred murine EAE.. Journal of Neuroimmunology.

[64] Cross AH, Misko TP, Lin RF, Hickey WF, Trotter JL (1994). Aminoguanidine, an inhibitor of inducible nitric oxide synthase, ameliorates experimental autoimmune encephalomyelitis in SJL mice.. Journal of Clinical Investigation.

[65] Cross AH, Ramsbottom MJ, Lyons J-A (2006). NOS2 regulates cytokine production and VLA-4 expression in experimental autoimmune encephalomyelitis.. Journal of Neuroimmunology.

[66] Dalton DK, Wittmer S (2005). Nitric-oxide-dependent and independent mechanisms of protection from CNS inflammation during Th1-mediated autoimmunity: evidence from EAE in iNOS KO mice.. Journal of Neuroimmunology.

[67] Kahl KG, Schmidt HH, Jung S, Sherman P, Toyka KV (2004). Experimental autoimmune encephalomyelitis in mice wiht a targeted deletion of the inducible nitric oxide synthase gene: increased T-helper 1 response.. Neuroscience Letters.

[68] Smith KJ, Lassmann H (2002). The role of nitric oxide in multiple sclerosis.. Lancet Neurology.

[69] Albarracin R, Eells JT, Valter K (2011). Photobiomodulation Protects the Retina from Light-Induced Photoreceptor Degeneration.. Invest Ophthalmol Vis Sci.

[70] Khalili K, White MK, Lublin F, Ferrante P, Berger JR (2007). Reactivation of JC virus and development of PML in patients with multiple sclerosis.. Neurology.

[71] Roberts JE (2000). Light and Immunomodulation.. Annals of the New York Academy of Sciences.

[72] Ribeiro MA, Albuquerque RL, Ramalho LM, Pinheiro AL, Bonjardim LR (2009). Immunohistochemical assessment of myofibroblasts and lymphoid cells during wound healing in rats subjected to laser photobiomodulation at 660 nm.. Photomedicine and Laser Surgery.

[73] Suryani S, Sutton I (2007). An interferon-gamma-producing Th1 subset is the major source of IL17 in experimental autoimmune encephalomyelitis.. Journal of Neuroimmunology.

[74] Waldburger KE, Hastings RC, Schaub RG, Goldman SJ, Leonard JP (1996). Adoptive transfer of experimental allergic encephalomyelitis after in vitro treatment with recombinant murine interelukin-12. Preferential expansion of interferon-gamma-producing cells and increased expression of macrophage-associated inducible nitric oxide synthase as immunomodulatory mechanisms.. American Journal of Pathology.

[75] Hickey WF, Hsu BL, Kimura J (1991). T-lymphocyte entry into the central nervous system.. Journal of Neuroscience Research.

[76] Male D, Pryce G, Hughes C, Lantos PL (1990). Lymphocyte migration into Brain modelled *in vitro*: control by lymphocyte activation, cytokines, and antigen.. Cellular Immunololgy.

[77] Fitzgerald M, Bartlett CA, Payne SC, Hart NS, Rodger J (2010). Near Infrared Light Reduces Oxidative Stress and Preserves Function in CNS Tissue Vulnerable to Secondary Degeneration Following Partial Transection of the Optic Nerve.. Journal of Neurotrauma.

